# A Humanities-Based Explanation for the Effects of Emotional Eating and Perceived Stress on Food Choice Motives during the COVID-19 Pandemic

**DOI:** 10.3390/nu12092712

**Published:** 2020-09-04

**Authors:** Wan Shen, Lucy M. Long, Chia-Hao Shih, Mary-Jon Ludy

**Affiliations:** 1 Food and Nutrition Program, Department of Public and Allied Health, Bowling Green State University, Bowling Green, OH 43403, USA; mludy@bgsu.edu; 2 Center for Food and Culture, 550 West Wooster St, Bowling Green, OH 43402, USA; LucyL@bgsu.edu; 3 Institute for Study of Culture and Society, Bowling Green State University, Bowling Green, OH 43403, USA; 4 Department of Psychiatry, University of Toledo, Toledo, OH 43606, USA; chiahao.shih@utoledo.edu

**Keywords:** perceived stress, food choice motives, emotional eating, COVID-19

## Abstract

Perceived stress affects emotional eating and food choices. However, the extent to which stress associates with food choice motives is not completely understood. This study assessed whether emotional eating mediates the associations between perceived stress levels and food choice motives (i.e., health, mood, convenience, natural content, price, sensory appeal, familiarities, weight control, and ethical concerns) during the Coronavirus Disease 2019 pandemic. A total of 800 respondents were surveyed in the United States in June 2020. Their perceived stress, emotional eating, and food choice motives were assessed by the Perceived Stress Scale, Dutch Eating Behavior Questionnaire, and Food Choice Questionnaire, respectively. Moderate to high levels of perceived stress were experienced by the majority (73.6%) of respondents. Perceived stress was significantly correlated with emotional eating (*r* = 0.26) as well as five out of nine food choice motives: mood (*r* = 0.32), convenience (*r* = 0.28), natural content (*r* = −0.14), price (*r* = 0.27), and familiarity (*r* = 0.15). Emotional eating was significantly correlated with four out of nine food choice motives: mood (*r* = 0.27), convenience (*r* = 0.23), price (*r* = 0.16), and familiarity (*r* = 0.16). The mediation analyses showed that emotional eating mediates the associations between perceived stress and five food choices motives: mood, convenience, sensory appeal, price, and familiarity. Findings were interpreted using theories and concepts from the humanities, specifically, folklore studies, ritual studies, and symbolic anthropology.

## 1. Introduction

Stress, anxiety, and depression are emotions associated with undesirable eating behaviors [1]; heightened levels of these emotions have been reported worldwide during the Coronavirus Disease 2019 (COVID-19) pandemic [2,3,4,5,6,7,8,9,10,11,12]. In January 2020, one week after the initial COVID-19 outbreak in Wuhan, China, a nationwide survey indicated that over 35% of Chinese people were experiencing peritraumatic distress related to COVID-19 [10]. Shortly after, the United States (US) faced its own COVID-19 outbreak and declared a state of emergency in March. With the huge disruption in daily routines and social interactions, the public experienced multiple stressors such as loneliness [4,13], fear of the disease [4], caregiver burden [4,14], financial pressure [4], food insecurity [4,15,16], and uncertainty about the future [4]. In April, a nationwide survey of US adults reported that 13.6% had symptoms associated with serious psychological distress; three times higher than in 2018 [6]. Likewise, COVID-19-related mental health concerns such as depression, anxiety, and post-traumatic stress disorder symptoms were prevalent among US young adults, affecting 43%, 45%, and 31% of this population, respectively [5].

The physical and emotional exhaustion from exposure to stress directly disrupts nutrition and eating patterns [2,17,18]. Although acute stress typically suppresses appetite [19,20], persistent stress signals the adrenal glands to release cortisol, which promotes appetite, the motivation to eat, and the consumption of palatable energy-dense foods that are high in calories, sugar, and fat [19,21,22,23]. Levels of other hormones such as ghrelin, insulin, and leptin are also influenced under chronic stress and greatly impact on satiety, appetite, and food choices [24]. Dietary intake of energy and fat is increased under stressed conditions [22,25,26] and stress-induced preferences for “comfort foods” that contain high amounts of added sugar and/or solid fat are common [27,28,29]. Positive associations between stress and intakes of soda, salty snacks, sweet cakes, and fast food are common. Likewise, negative associations between stress and intakes of fresh fruits and vegetables have been demonstrated [30,31,32,33]. Early research during the COVID-19 pandemic suggests changes in dietary behavior and food choices [34,35]: consumption of pasta, flour, and frozen food has increased, while consumption of fresh foods has decreased [36,37,38,39].

Food choice motives are important factors that drive people in making food selections. These motives include social, cultural, aesthetic, political, and contextual factors as well as the nutritional value of the food [40,41]. Commonly recognized factors that influence consumer food choice decisions include health [42,43,44,45,46,47,48], mood [43,46,49], convenience [43,47,49,50], natural content [42,43,44,45,46,47,48], price [43,46,47,50,51], sensory appeal [42,43,47], familiarity [45,46,49,51], weight control [46], and ethical concerns/agreement [46,47].

Emotional eating is defined as eating in response to negative emotions (e.g., perceived stress) without following internal hunger cues [52]. Emotional eaters report greater intake of sweet, fatty, and salty snack foods [53,54]. Studies have indicated that emotional eating is positively associated with body mass index [55,56,57,58,59], stress [57,60,61], and depression [62,63,64]. In most studies, females were more influenced by emotional eating, leading to greater weight gain compared to males [55,59,62].

**Figure 1:**
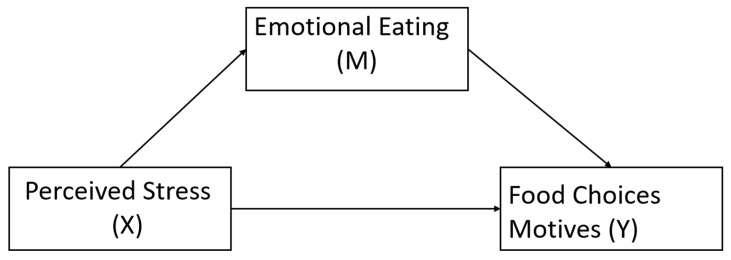
Proposed mediation model of perceived stress on food choice motives.

Taken together, stress affects people’s food choices and eating behaviors. Yet, to the best of our knowledge, no study has explored the relationships between perceived stress, emotional eating, and food choice motives with the statistical framework of mediation. Therefore, the current study aimed to test whether emotional eating mediates the relationships among perceived stress and food choice motives (i.e., health, mood, convenience, natural content, price, sensory appeal, familiarities, weight control, and ethical concerns) during the COVID-19 pandemic. Participants’ perceived stress levels, eating behaviors, and food choice motives were assessed in June 2020, two months after the “stay-at-home” order was implemented in at least 42 states in the US. Preliminary research suggests this prolonged homestay may be negatively impacting mental health [4,35,65] and dietary behavior changes [35,65]. We hypothesized that the relationship between perceived stress and food choice motives would be mediated by emotional eating (Figure 1).

To contextualize findings, we applied theories and concepts from the humanities, specifically, folklore studies (folkloristics), ritual studies, and symbolic anthropology. Humanities disciplines study the ways in which people have attempted to make sense of their lives and the world around them. They explore the meanings individuals and groups have constructed and attached to events, actions, things, and other beings [63,64]. As such, they offer a lens for interpreting the intangible aspects of our lives and help us understand the cultural and social forces that shape our choices. This study draws upon two concepts from the humanities as interpretive lens. First, the humanities approach food in general as a dynamic and constructed medium through which individuals and groups express and negotiate their identities, relationships, worldview, and values. Therefore, the meanings attached to particular foods, including those categorized as “comfort foods” [66], are also dynamic and fluid, reflecting cultural, social, historical, political, economic, ethical/moral, and environmental forces as well as an individual’s personal history, values, and personality [63,67,68,69]. Secondly, activities around food can be approached as rituals (i.e., recurring symbolic events). As such, the concept of liminality suggests a lens for viewing our experiences of the COVID-19 pandemic as a “time out of time,” that is, a stage in between a previous order and a new one to come [70,71,72]. During such times, people are not expected to behave as they did previously and can explore new rules, including those shaping their food choices. Motivations behind those choices are perhaps reflecting the uncertainties created by the pandemic.

## 2. Materials and Methods

### 2.1. Study Design

Adults living in the US were recruited to participate in this cross-sectional study regarding food choices during the COVID-19 pandemic. Data were collected from 1 to 27 June 2020 using the Qualtrics online survey system (Qualtrics, Provo, UT, USA). Eligibility criteria included being at least 18 years old and residing in the US at the time of the survey. A total of 1001 responses were initially collected. However, 201 were excluded due to a response completion of less than 30% (*n* = 124), completion time of less than 8 min (*n* = 76), or implausible responses (*n* = 1). All participants were provided informed consent forms using processes approved by the Office of Research Compliance at Bowling Green State University (BGSU IRB 1606044, approved in May 2020). All subjects gave their informed consent for inclusion before they participated in the study. Participants with completed responses were entered into a raffle for 1 of 50 Amazon gifts cards (valued at $20).

### 2.2. Demographics

Demographic information included self-reported gender, age, weight, height, education level, ethnicity, and household income. Body mass index (kg/m^2^) was calculated based on self-reported weight and height. A summary of the demographic data is presented in Table 1.

### 2.3. Assessment of Perceived Stress

Perceived stress was assessed using the validated 10-question Perceived Stress Scale [73], a classic stress assessment tool developed to assess feelings and thoughts in the past month, for example “In the last month, how often have you been upset because of something that happened unexpectedly.” Participants were asked to select the frequency of occurrence on a five-point scale ranging from “never” (scored 0) to “very often” (scored 4); four questions were reversely scored. After totaling scores from the 10 questions, participants with scores between 0 and 13 were identified as “Low Stress,” between 14 and 26 were “Moderate Stress,” and between 27 to 40 were “High Stress.” Cronbach’s alpha for the current sample was 0.81, demonstrating good internal consistency.

### 2.4. Assessment of Food Choice Motives

Food choice motives were assessed using the validated 36-item Food Choice Questionnaire [47], which specifically asked about factors that influenced food choices on a typical day. A total of nine food choice factors were assessed from the instrument: health (e.g., if the chosen food is nutritious), mood (e.g., if the chosen food helped with relaxation), convenience (e.g., if the chosen food is easy to access or prepare), sensory appeal (e.g., if the chosen food tastes good), natural content (e.g., if the chosen food contains no additives), price (e.g., if the chosen food is cheap or good value for money), weight control (e.g., if the chosen food is low in calories or fat content), familiarity (e.g., if the chosen food is familiar or frequently consumed), and ethical concern (e.g., if the chosen food is produced and distributed in a manner perceived as sustainable or ethical). Participants were asked to select the importance of each factor on a five-point scale ranging from “unimportant” (scored 1) to “very important” (scored 5). Averaged scores from subcategories were calculated. Cronbach’s alpha for the current sample was 0.91, demonstrating excellent internal consistency.

### 2.5. Assessment of Emotional Eating

Emotional eating was assessed using the validated 33-item Dutch Eating Behavior Questionnaire [74], a classic instrument to assess three eating behaviors: emotional eating, restrained eating, and external eating. Participants responded to questions on a five-point Likert-type scale ranging from “never” (scored 1) to “very often” (scored 5). Scores were averaged from emotional eating related questions. Other subscales from this questionnaire (i.e., restrained and external eating) were not the focus of this project. Cronbach’s alpha for the current sample was 0.93, demonstrating excellent internal consistency.

### 2.6. Statistical Analysis

We examined whether associations between perceived stress and nine food choice motives were mediated by emotional eating by fitting a simple mediation model for each of the food choice motives. Data were expressed as mean ± standard deviation for continuous variables or number (percentage) for categorical variables. Pearson correlations were used to evaluate linear relationships between perceived stress, emotional eating, and the nine food choice motives. The Bonferroni correction was applied to avoid alpha inflation. Ordinary least square regression was used to test the proposed mediation model, where gender, age group, education level, ethnicity, and annual household income were entered into the model as covariates. In this mediation model, perceived stress scores represented the independent variable, emotional eating scores represented the mediator, and each of the nine food choice motives scores represented the dependent variable. For this simple mediation model, significance of the direct and indirect effects was tested with 95% confidence intervals (CI), which were established by utilizing non-parametric bootstrapping with 5000 samples. These effects were considered statistically significant if 0 fell outside of the 95% CI. List-wise deletion was applied when missing values occurred in the above analyses (i.e., *n* = 769 and 767 for correlation and mediation analyses, respectively). All statistical analyses were performed using SPSS version 23 (IBM Corporation, Armonk, NY) and the “PROCESS” macro for SPSS [75]. Statistical significance level was set at α = 0.05, two-tailed.

## 3. Results

### 3.1. Demographics

Data from 800 respondents was included in the study (Table 1). The majority of the participants were female (83.0%), white (77.5%), aged between 25−44 (42.0%), and had a bachelor’s degree or higher (64.4%). The average of the perceived stress scores was 18.27 ± 7.34 (Table 2), and the majority (73.6%) reported moderate to high levels of perceived stress (scored between 14 to 40) (Table 1).

### 3.2. Correlation Analysis

The averaged scores for perceived stress, emotional eating and the nine food choice motives are shown in Table 2. Bivariate correlations between variables of interest are also included in Table 2. Perceived stress was significantly correlated with emotional eating as well as five out of nine food choice motives. These include mood, convenience, natural content, price, and familiarity (ps < 0.05, Bonferroni-corrected). Emotional eating was significantly correlated with four out of nine food choice motives. These include mood, convenience, price, and familiarity (ps < 0.05, Bonferroni-corrected). The majority of the nine food choice motives were significantly correlated with each other at weak to moderate levels. These ranged from 0.12 for price and ethical concern to 0.66 for health and natural content (ps < 0.05, Bonferroni-corrected). Exceptions included health and price, convenience and natural content, natural content and price, as well as natural content and familiarity, which were not correlated (ps > 0.05, Bonferroni-corrected).

### 3.3. Mediation Analysis

**Figure 2:**
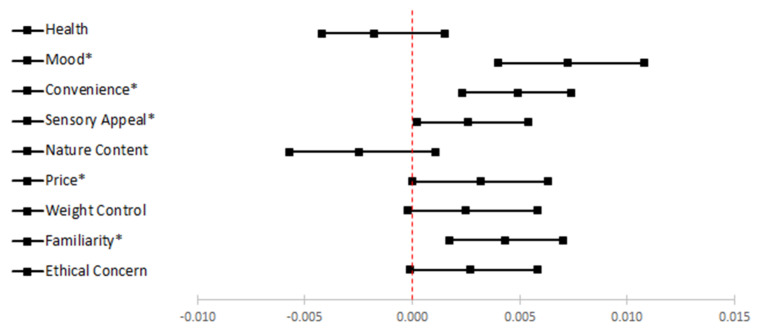
Forest plot for the mediation results.

We tested whether the associations between perceived stress and food choice motives were mediated by emotional eating; each of the nine food choice motives was tested separately (Figure 1). Model estimations are summarized in Table 3; 95% CIs for indirect effects are presented in Figure 2. Overall, emotional eating mediated the associations between perceived stress and five out of nine food choice motives including mood, convenience, sensory appeal, price, and familiarity.

Health. The total effect between perceived stress and health was not significant (βc = −0.0076, SE = 0.0044, 95% CI [−0.0163, 0.0011]). In addition, the direct effect between perceived stress and health, while holding emotional eating levels constant, was not statistically significant (βc’ = −0.0058, SE = 0.0046, 95% CI [−0.0148, 0.0032]). Furthermore, the indirect effect of emotional eating in the association between perceived stress and health did not reach statistical significance (βab = −0.0018, SE = 0.0014, 95% CI [−0.0042, 0.0015]).

Mood. The total effect between perceived stress and mood was significant (βc = 0.0401, SE = 0.0044, 95% CI [0.0314, 0.0488]). In addition, the direct effect between perceived stress and mood, while holding emotional eating levels constant, was significant (βc’ = 0.0329, SE = 0.0045, 95% CI [0.0241, 0.0418]). Moreover, the indirect effect of emotional eating in the association between perceived stress and mood reached statistical significance (βab = 0.0072, SE = 0.0018, 95% CI [0.0040, 0.0108]), completely standardized indirect effect = 0.0562, 95% CI [0.0317, 0.0839]). Thus, higher perceived stress was associated with greater tendency toward emotional eating which, in turn, was positively associated with mood-based food choice.

Convenience. The total effect between perceived stress and convenience was significant (βc = 0.0281, SE = 0.0038, 95% CI [0.0207, 0.0355]). In addition, the direct effect between perceived stress and mood, while holding emotional eating levels constant, was significant (βc’ = 0.0233, SE = 0.0038, 95% CI [0.0157, 0.0308]). Moreover, the indirect effect of emotional eating in the association between perceived stress and convenience reached statistical significance (βab = 0.0049, SE = 0.0013, 95% CI [0.0023, 0.0074]), completely standardized indirect effect = 0.0458, 95% CI [0.0225, 0.0695]). Thus, higher perceived stress was associated with greater tendency toward emotional eating, which, in turn, was positively associated with convenience-based food choice.

Sensory appeal. The total effect between perceived stress and sensory appeal was not significant (βc = 0.0039, SE = 0.0041, 95% CI [−0.0042, 0.0119]). In addition, the direct effect between perceived stress and sensory appeal, while holding emotional eating levels constant, was not statistically significant (βc’ = 0.0012, SE = 0.0042, 95% CI [−0.0071, 0.0095]). However, the indirect effect of emotional eating in the association between perceived stress and sensory appeal reached statistical significance (βab = 0.0026, SE = 0.0013, 95% CI [0.0002, 0.0054]), completely standardized indirect effect = 0.0237, 95% CI [0.0021, 0.0480]). Thus, higher perceived stress was associated with greater tendency toward emotional eating, which, in turn, was positively associated with sensory appeal-based food choice.

Natural content. The total effect between perceived stress and natural content was significant (βc = −0.0196, SE = 0.0055, 95% CI [−0.0304, −0.0088]). In addition, the direct effect between perceived stress and natural content, while holding emotional eating levels constant, was statistically significant (βc’ = −0.0171, SE = 0.0057, 95% CI [−0.0282, −0.0152]). However, the indirect effect of emotional eating in the association between perceived stress and natural content did not reach statistical significance (βab = −0.0025, SE = 0.0017, 95% CI [−0.0057, 0.0011]).

Price. The total effect between perceived stress and price was significant (βc = 0.0329, SE = 0.0048, 95% CI [0.0235, 0.0423]). In addition, the direct effect between perceived stress and price, while holding emotional eating levels constant, was significant (βc’ = 0.0297, SE = 0.0050, 95% CI [0.0200, 0.0395]). Moreover, the indirect effect of emotional eating in the association between perceived stress and mood reached statistical significance (βab = 0.0032, SE = 0.0016, 95% CI [0.0000, 0.0063]), completely standardized indirect effect = 0.0229, 95% CI [0.0003, 0.0451]). Thus, higher perceived stress was associated with greater emotional eating, which, in turn, was positively associated with price-based food choice.

Weight control. The total effect between perceived stress and weight control was not significant (βc = −0.0050, SE = 0.0051, 95% CI [−0.0150, 0.0051]). In addition, the direct effect between perceived stress and weight control, while holding emotional eating levels constant, was not statistically significant (βc’ = −0.0075, SE = 0.0053, 95% CI [−0.0179, 0.0029]). Furthermore, the indirect effect of emotional eating in the association between perceived stress and weight control did not reach statistical significance (βab = 0.0025, SE = 0.0015, 95% CI [−0.0002, 0.0058]).

Familiarity. The total effect between perceived stress and familiarity was significant (βc = 0.0191, SE = 0.0044, 95% CI [0.0104, 0.0278]). In addition, the direct effect between perceived stress and familiarity, while holding emotional eating levels constant, was significant (βc’ = 0.0148, SE = 0.0046, 95% CI [0.0058, 0.0238]). Moreover, the indirect effect of emotional eating in the association between perceived stress and familiarity reached statistical significance (βab = 0.0043, SE = 0.0014, 95% CI [0.0017, 0.0070], completely standardized indirect effect = 0.0354, 95% CI [0.0140, 0.0571]). Thus, higher perceived stress was associated with a greater tendency toward emotional eating, which, in turn, was positively associated with familiarity-based food choice.

Ethical concern. The total effect between perceived stress and ethical concern was not significant (βc = −0.0050, SE = 0.0051, 95% CI [−0.0150, 0.0050]). In addition, the direct effect between perceived stress and ethical concern, while holding emotional eating levels constant, was not statistically significant (βc’ = −0.0078, SE = 0.0053, 95% CI [−0.0181, 0.0026]). Furthermore, the indirect effect of emotional eating in the association between perceived stress and ethical concern did not reach statistical significance (βab = 0.0027, SE = −0.0015, 95% CI [−0.0001, 0.0058]).

## 4. Discussion

In this study, the mediating role of emotional eating on the relationships between perceived stress and food choice motives (i.e., health, mood, convenience, natural content, price, sensory appeal, familiarities, weight control, and ethical concerns) was examined. Findings from the mediation analyses support that perceived stress contributed to several food choices motives through emotional eating. Specifically, the perception of stress was positively associated with emotional eating, and the greater tendency toward emotional eating, the greater the desire to choose foods based on motives such as mood, convenience, sensory appeal, price, and familiarity.

To our knowledge, this is the first study that has explored the relationships among perceived stress, emotional eating, and food choice motives. Existing studies have demonstrated that perceived stress influences specific food choices [22,27,31,33,37,61,76,77], yet, it is important to reveal the underlying reason(s) that food choice determinants are affected under stress so that proper intervention can be developed. This is especially true during the COVID−19 pandemic.

Motivations for food choices always go beyond the nutritional value of the food. The individual’s cultural, social, and personal identities and experiences shape their concepts of what can be considered food, what is tasty and healthy food, and what are the appropriate ways to procure, prepare, and consume it [63,67,68,78,79,80,81,82]. The symbolic meanings attached to food oftentimes shape an individual’s reaction to that food, influencing their choices [40,41,63,83,84,85,86,87,88]. Furthermore, the specific circumstances and contexts motivate the specific choices being made. This can reflect the social relationships held or aspired to by an individual [40,89,90,91].

One source of stress related to food choice is that the pandemic has affected the food system itself, disrupting the usual food chain from production to distribution to consumption and even disposal. The seemingly simply act of purchasing an apple at a supermarket requires a complex network of growers, harvesters, packagers, and distributors. These networks tend to be invisible, so that we are usually able to make our individual food choices without recognizing that system—or the individuals involved in it. Now, because of the pandemic, we are forced to acknowledge that disruptions in the food system in a seemingly distant place can impact us. For example, COVID-19 among workers in a pork processing plant translates into a potential shortage of pork products in supermarkets [92]; quarantines result in a lack of individuals to harvest crops, resulting in those crops no longer being easily available [93]. These disruptions challenge our feelings of security; after all, we have been assured by the contemporary industrial food system that we are now immune to food shortages, however temporary.

Emotional eating disinhibits self-control of dieting or “restraint eating” [94]. The tendency to eat in response to perceived stress is typical and linked to weight gain [95]. For example, emotional eating mediated the association between elevated depressive symptoms and increased BMI among women living in the greater New Orleans areas after Hurricane Katrina [96]. Similar results were found among people living in Spain after the 2008 banking crisis [97]. The increase in adiposity might be explained through increased consumption of “comfort food” [29,98,99].

The concept of “comfort food” was introduced by the American psychologist, Joyce Brothers, who used it to explain the rise of obesity in the US in the 1960s [66]. It is used now as a marketing category in the food industry that gives individuals permission to consume the “bad foods” [62] that are in normal times considered detrimental to one’s physical health or body image [66]. According to foundational research by Julie Locher, such foods were consumed in times of stress, relieving that stress through one of four characteristics: nostalgia, indulgence, convenience, or physical comfort [100,101].

“Presenting a food as comfort food, then, means that the usual concerns for health, nutrition, convenience, expense, environmental sustainability or other factors motivating our food choices can be suspended while we focus on the emotional and nurturing aspects of the food [66].”

Such foods represent the opposite of “restraint eating” and are being emphasized during the current period of stress caused by the pandemic.

In this study, emotional eating mediated the associations between perceived stress and food choice motives including mood, convenience, sensory appeal, price, and familiarity: the greater the tendency toward emotional eating, the higher the desire to choose foods based on the five food choice motives mentioned. A number of pragmatic explanations for these factors can be identified along with emotional and social ones. As discussed above, “comfort food” is viewed as important for relieving stress and improving mood in general.

Convenience as a motivator reflects pragmatic concerns. During the pandemic, daily routines have changed dramatically: many individuals are working from home and discovering that such work is more time consuming than expected [102]. The lack of office space and the temporal and spatial distinction between work life and personal life may make it difficult for some individuals to schedule their days productively [103,104]. Additionally, unexpected homeschooling and lack of childcare challenge numerous working parents, disrupting their quantity and quality of work [105]. Choosing and preparing food can be an added burden for these individuals, particularly when it might now need to be achieved three or more times a day [106,107,108]. Foods that are convenient to prepare, consume, and clean up offer a solution to these dilemmas. Convenience was also one of the characteristics of “comfort food” identified by Locher and other researchers [100,101].

Sensory appeal, the taste and presentation of food, may function as a motivator for emotional eating by offering psychological escape and distraction [65]. Aesthetic objects and activities engage our senses, focusing our attention on the experience at hand and taking our minds off of other issues, including the current pandemic situation [109]. Creating a pleasing dish, furthermore, gives us a sense of accomplishment, adding to our sense of self-esteem and creating positive emotions [110,111]. It can also enable us to feel that we still have some control over our lives during a time in which control over many other aspects is uncertain [112,113,114]. The massive popularity of baking in the U.S. that emerged with the pandemic can perhaps be explained in this way [115].

Price as a food choice motive reflects the uncertainty about work and financial futures, and the sense of impending precarity that many are experiencing. Financial burdens caused by job losses and shut-downs due to the pandemic [116] may necessitate food choices to be more affordable, encouraging consumers to cut back on food purchases and save money during the pandemic [117,118]. Money for food might also be rechanneled for other expenditures instead, such as medicine or rent [119,120].

The emphasis on familiarity of foods during the pandemic can be tied to both practical needs as well as emotional and psychological ones [121,122,123,124,125,126]. On the one hand, we might need to make sure people will eat the food being served. We know what to expect with familiar dishes, thus, we can gauge whether or not they will satisfy hunger and nutritional needs [40,63,66,101]. On a more emotional level, though, they are tied to our identities. Familiar foods can bring up warm nostalgic feelings that can comfort us [66]. They remind us of who we are, grounding our personal histories in larger historical movements and reminding us of continuity when the future is uncertain [66,127,128]. They can also offer participation in social groups built around our various identities, offering social networks during a time when many are experiencing isolation [40,63,83,89].

The concept of liminality offers a lens for further interpreting all of these food choice motives. Liminality occurs during rites of passage when an individual or a group moves from one stage of life to another. During such times, the usual rules are suspended. The rules of the earlier stage no longer apply, but participants are not yet expected to adhere to the rules of the new stage. In this “time out of time,” they might make mistakes, behave inappropriately, and fail in other ways without censure or punishment [70,71,72]. The pandemic can be viewed as a liminal stage in which the usual rules behind food choices are suspended. Individuals concerned about weight might decide not to worry about that during this time and thus consume foods they normally would feel were too fattening [129,130]. Concerns about nutrition, natural content, and sustainability might similarly be set aside. Part of the difficulty, though, is that none of us know what the next stage—the new normal—will actually be and how that is going to affect our food choices. Predictions about disruptions in the industrial food system itself as well as fears about economic futures and the on-going challenges of being in public spaces mean that the new rules are uncertain. Interpreting this time as one of liminality helps connect the stress caused by the pandemic with food choices: the uncertainty of the future, not only in terms of food but of everyday life in general, is suspending the usual rules.

## 5. Interventions

Acknowledging the food choice motives led by emotional eating under stress helps us to understand the why and how the food choices being made at this time. Developing proper interventions among vulnerable populations (such as females or people with BMI ≥30 or/and that have chronic medical conditions) in the community is needed. Interestingly, high BMI is a well-known risk factor for COVID-19 [131,132,133], and in certain regions, prevalence of COVID-19 is higher in women [134]. Relaxation helps relieve emotional eating [135]. Nutrition counseling focusing on choosing healthier foods that are easily prepared, foods that are readily available, and smaller serving sizes might help emotional eaters with weight concerns. Although healthier options such as fresh fruits, vegetables and whole grain products tend to cost more, encouraging more home production and preparation of foods can relieve some financial burden [136]. Additionally, substituting fresh with frozen or canned foods, which can also be very healthy, can be encouraged. Education about the nature of comfort food as both a marketing category and an approach to food that is tied to each individual’s personal history can also help people make healthier choices [40,137]. Similarly, encouraging the exploration of the relationship between food and cultural identity can make consumers more reflective of the meanings certain foods hold for them. Such exploration may increase awareness of the potential for social networks and community building offered by food, enabling people to address the isolation experienced by many Americans in general that is heightened during this time. Similar education about the values of attending to the sensory properties of food could also encourage consumers to focus on the potential aesthetic experience of any food being consumed, including mundane, everyday types of foods not usually perceived as “gourmet” and worthy of attention.

Furthermore, we can encourage consumers to see this time period as one of liminality, one in which we can formulate new rules for our food choices. It is an opportunity to renew older practices and relationships that may have been put aside, such as family meals and recipe sharing and other activities that help people reconnect to their culture heritage, traditions, and identities. It is also an opportunity to develop new traditions that increase social interaction, sharing of knowledge and skills, and feelings of belonging. Virtual dinner parties, birthday celebrations, “happy hour quarantinis,” and similar events offer companionship and a sense of connectedness that can offset the stress that leads to unhealthy emotional eating.

## 6. Strengths and Limitations

The strengths of the study include a large sample size to ensure adequate power to conduct analyses. Additionally, all questionnaires used in the study were previously validated. The pandemic hit us abruptly earlier in the year; it has not been well understood how emotional eating and food choice motives are being affected under the COVID-19. Perspectives from humanities disciplines provide a useful lens that contextualizes the interpretation of the findings.

There are limitations to the study. First, the study was cross-sectional, thus, the causal relationship cannot be inferred among the variables. Second, all data, including weight and height, were self-reported in the study. Under—or over—reporting might exist. Third, the sample may not be representative as the majority responses were from highly educated, white, female participants. Additionally, despite significant correlation among perceived stress, emotional eating, and some of the food choice motives as well as mediational links among these variables, it should be noted that the effect sizes were small, and therefore, the implications of the findings (as discussed in the previous section) should be carefully considered. Furthermore, using humanity theories as an interpretative lens helps us identify some of the intangibles associated with food choice motivations. Some scholars, however, may find this lens too descriptive and conceptual. While it is possible to count and measure the meanings people may attach to their food choices, humanities disciplines tend to focus on interpretation of data rather than the methodologies of collecting that data. These interpretations then open up new perspectives on the complexities and nuances of human behavior. Moreover, the humanities tend to not offer specific solutions to issues, but try to shed light on those issues so that individuals and groups can then develop strategies that are compatible with their own cultures, belief systems, and situations. Interventions from a humanities perspective, therefore, emphasize expanding our knowledge and broadening our understandings of the world we have created. Such expansion can be measured, but tends towards the intangible; thus, measurement of efficacy is difficult. It might be, however, that it is these intangibles that need to be addressed to identify why people behave the way they do in relationship to food and to affect change in that behavior.

## Figures and Tables

**Table 1:** Demographics.

	*n* (%)
**Gender**	
Female	664 (83.00%)
Male	130 (16.25%)
Transgender	6 (0.75%)
**Age**	
18 – 24	60 (7.50%)
25 – 34	167 (20.88%)
35 – 44	169 (21.11%)
44 – 54	145 (18.13%)
55 – 64	67 (8.4%)
≥65	192 (24.00%)
BMI *	
<18.5	18 (2.29%)
18.5 − 24.9	273 (34.73%)
25 − 29.9	213 (27.10%)
≥30	282 (35.88%)
**Education**	
High school or below	132 (16.50%)
Associate degree	95 (11.88%)
Bachelor degree	172 (21.50%)
Master’s/Doctorate degree	343 (42.88%)
Other	58 (7.25%)
**Ethnicity**	
White	620 (77.50%)
Hispanic or Latino	28 (3.50%)
Black or African American	46 (5.75%)
American Indian or Alaska Native	9 (1.13%)
Asian	80 (10.00%)
Native Hawaiian or Pacific Islander	1 (0.13%)
Other	16 (2.00%)
**Household income**	
$0–$24,999	142 (17.75%)
$25,000–$49,999	154 (19.25%)
$50,000–$74,999	174 (21.75%)
$75,000–$99,999	120 (15.00%)
$100,000–$149,000	117 (14.63%)
≥$150,000	93 (11.63%)
**Perceived Stress**	
Low stress	211 (26.38%)
Moderate stress	473 (59.13%)
High stress	116 (14.50%)

* Missing 14 data points due to implausible self-reported height or weight.

**Table 2:** Correlations for all outcome measured.

Measures	1	2	3	4	5	6	7	8	9	10	11	Mean (SD)
Perceived Stress (1)	-	0.2*	−0.07	0.3 *	0.2 *	0.03	−0.14 *	0.2 *	−0.05	0.1 *	−0.04	18.27 (7.34)
Emotional Eating (2)	-	-	−0.09	0.2 *	0.2 *	0.1	−0.09	0.1 *	0.05	0.1 *	0.07	2.84 (1.17)
Health (3)	-	-	-	0.2 *	0.1 *	0.3 *	0.66 *	0.06	0.5 *	0.1 *	0.4 *	3.30 (0.91)
Mood (4)	-	-	-	-	0.4 *	0.5 *	0.13 *	0.1 *	0.1 *	0.4 *	0.3 *	2.97 (0.94)
Convenience (5)	-	-	-	-	-	0.3 *	0.03	0.4 *	0.1 *	0.4 *	0.1 *	3.48 (0.78)
Sensory Appeal (6)	-	-	-	-	-	-	0.24 *	0.1 *	0.2 *	0.4 *	0.3 *	3.42 (0.82)
Natural Content (7)	-	-	-	-	-	-	-	−0.04	0.5 *	0.08	0.5 *	2.78 (1.12)
Price (8)	-	-	-	-	-	-	-	-	0.1 *	0.2 *	0.1 *	3.56 (1.02)
Weight Control (9)	-	-	-	-	-	-	-	-	-	0.1 *	0.3 *	2.63 (1.02)
Familiarity (10)	-	-	-	-	-	-	-	-	-	-	0.3 *	2.91 (0.90)
Ethical Concern (11)	-	-	-	-	-	-	-	-	-	-	-	2.21 (1.04)

After list-wise deletion, data from 769 respondents were analyzed by correlation. The Pearson correlation test was used to evaluate linear relationships between outcome variables. *r* values are shown in the table. * *p <* 0.05, Bonferroni corrected.

**Table 3:** Model coefficient estimations for mediation analyses.

				Consequent											
	M (Emotional Eating)			Y_1_ (Health)			Y_2_ (Mood)			Y_3_ (Convenience)			Y_4_ (Sensory Appeal)		
Antecedent	Coeff.	SE	*p*	Coeff.	SE	*p*	Coeff.	SE	*p*	Coeff.	SE	*P*	Coeff.	SE	*p*
X (Perceived Stress)	0.043	0.006	<0.001	−0.006	0.005	0.208	0.033	0.005	<0.001	0.023	0.005	<0.001	0.001	0.004	0.775
M (Emotional Eating)	-	-	-	−0.042	0.028	0.144	0.168	0.028	<0.001	0.139	0.024	<0.001	0.062	0.026	0.018
Constant	2.356	0.267	<0.001	3.072	0.219	<0.001	1.694	0.215	<0.001	2.198	0.242	<0.001	3.181	0.202	<0.001
	R^2^ = 0.084			R^2^ = 0.058			R^2^ = 0.159			R^2^ = 0.109			R^2^ = 0.025		
	F(6760) = 11.646, *p* < 0.001			F(7759) = 6.703, *p* < 0.001			F(7759) = 20.462, *p* < 0.001			F(7759) = 13.219, *p* < 0.001			F(7759) = 2.77, *p* = 0.008		
	Consequent														
	Y_5_ (Natural Content)			Y_6_ (Price)			Y_7_ (Weight Control)			Y_8_ (Familiarity)			Y_9_ (Ethical Concern)		
Antecedent	Coeff.	SE	*p*	Coeff.	SE	*p*	Coeff.	SE	*p*	Coeff.	SE	*P*	Coeff.	SE	*p*
X (Perceived Stress)	−0.017	0.006	0.003	0.03	0.005	<0.001	−0.008	0.005	0.157	0.015	0.005	0.001	−0.008	0.005	0.142
M (Emotional Eating)	−0.06	0.035	0.09	0.074	0.031	0.016	0.059	0.033	0.072	0.101	0.028	<0.001	0.064	0.033	0.049
Constant	2.813	0.271	<0.001	3.6	0.237	<0.001	2.34	0.252	<0.001	2.573	0.218	<0.001	1.75	0.252	<0.001
	R^2^ = 0.06			R^2^ = 0.132			R^2^ = 0.025			R^2^ = 0.049			R^2^ = 0.059		
	F(7759) = 6.841, *p* < 0.001			F(7759) = 16.506, *p* < 0.001			F(7759) = 2.78, *p* = 0.007			F(7759) = 5.607, *p* < 0.001			F(7759) = 6.815, *p* < 0.001		

After list-wise deletion, data from 769 respondents were analyzed by mediation. Coeff. = unstandardized coefficient for each path in the mediation analyses. SE = standard error.

## Abbreviations

United States	(US)
Coronavirus Disease 2019	(COVID-19)
confidence intervals	(CI)
